# Robotics-assisted visual-motor training influences arm position sense in three-dimensional space

**DOI:** 10.1186/s12984-020-00727-w

**Published:** 2020-07-14

**Authors:** Bulmaro A. Valdés, Mahta Khoshnam, Jason L. Neva, Carlo Menon

**Affiliations:** 1 Menrva Research Group, Schools of Mechatronic System and Engineering Science, Simon Fraser University, Metro Vancouver, BC Canada; 2 Université de Montréal, École de kinésiologie et des sciences de l’activité physique, Faculté de médecine, Montréal, QC Canada; 3 Centre de recherche de l’institut universitaire de gériatrie de Montréal, Montréal, QC Canada

**Keywords:** Kinesthesia, Position sense, Proprioception, Arm position training, Robotic rehabilitation, Upper-limb

## Abstract

**Background:**

Performing activities of daily living depends, among other factors, on awareness of the position and movements of limbs. Neural injuries, such as stroke, might negatively affect such an awareness and, consequently, lead to degrading the quality of life and lengthening the motor recovery process. With the goal of improving the sense of hand position in three-dimensional (3D) space, we investigate the effects of integrating a pertinent training component within a robotic reaching task.

**Methods:**

In the proof-of-concept study presented in this paper, 12 healthy participants, during a single session, used their dominant hand to attempt reaching without vision to two targets in 3D space, which were placed at locations that resembled the functional task of self-feeding. After each attempt, participants received visual and haptic feedback about their hand’s position to accurately locate the target. Performance was evaluated at the beginning and end of each session during an assessment in which participants reached without visual nor haptic feedback to three targets: the same two targets employed during the training phase and an additional one to evaluate the generalization of training.

**Results:**

Collected data showed a statistically significant [39.81% (p=0.001)] reduction of end-position reaching error when results of reaching to all targets were combined. End-position error to the generalization target, although not statistically significant, was reduced by 15.47%.

**Conclusions:**

These results provide support for the effectiveness of combining an arm position sense training component with functional motor tasks, which could be implemented in the design of future robot-assisted rehabilitation paradigms to potentially expedite the recovery process of individuals with neurological injuries.

## Introduction

Recent technological advancements have led to the increasing use of robotics in various clinical applications, ranging from patient monitoring systems to complex robotic platforms for minimally invasive surgery [1]. For the particular case of motor rehabilitation, robotic technology has been employed for diverse tasks, such as monitoring limbs’ movements, characterizing the level of impairment of individuals after neurological injury, and assisting with motor training as complementary tools to conventional physiotherapy [2, 3]. In addition, robotic platforms have been introduced as potential tools to quantitatively evaluate and document progress during motor recovery [4].

Proprioception, which is arguably defined as the awareness of the position and movements of limbs [5], has a functional role in performing activities of daily living [6]. It has been shown that the aging process negatively affects this capability to some degree [7]. Moreover, neurological conditions, such as stroke, could significantly degrade an individual’s ability to locate their limbs in space and to detect passive movements imposed on their joints [8]. Although proprioception is considered to have a key role in the motor recovery process [9, 10], there are no current standard rehabilitation protocols for proprioceptive training [11]. Nevertheless, robotic platforms provide a viable alternative to objectively evaluate proprioception and to implement training protocols to improve this sense [4, 11].

Several studies have addressed robotic assessment and training of proprioception for the upper limbs. Dukelow et al. used a KINARM exoskeleton robotic device (BKIN Technologies Ltd., Kingston, Ontario) to quantify arm position sense during a bimanual arm position matching task and to objectively assess motor performance during a visually-guided reaching task [12]. These assessments were completed in the transverse plane, and it was shown that the performance during such tasks correlates with determining the level of independence in carrying out activities of daily living and can potentially assist with diagnosing the level of motor impairment [12]. Gordon et al. employed a platform consisting of a horizontal digitizing tablet and a computer screen to characterize how reaching to different locations in the transverse plane is impaired in individuals with proprioceptive deficits [13]. The same group subsequently reported that providing visual feedback from hand position on a screen or viewing the limb improves reaching accuracy, however, knowledge of results, provided as a delayed visual feedback of the hand path at the end of each trial, was not found as effective in increasing reaching accuracy [14]. Another series of studies employed haptic feedback in the form of a robot-generated force field to assist individuals with reaching to several targets in the transversal plane [10, 15–18]. In these studies, which included both uni- and bi-manual paradigms, the level of force assistance was adjusted based on individuals’ motor function and their performance, and it was shown that such a protocol could improve the sense of limb position and movement, but retainment of gained benefits for stroke individuals might also be affected by the level of stroke-related impairment [10]. Proprioceptive training through delayed visual feedback in a virtual reality platform was addressed in [19, 20]. In this regard, individuals with stroke were asked to transfer objects in a virtual environment to a predefined target without visual feedback of the location of their hands. Hand cursor and target location were displayed after each trial and subsequently were hidden so the participants could have another chance at reaching to the same target. It was reported that such a protocol that relied mostly on proprioceptive information had a significant positive impact on the limb position sense [19, 20]. In a different study, performance feedback was provided in the form of haptic and vibro-tactile feedback, with reported benefits of proprioceptive training of the wrist to reach a certain degree of movement in wrist flexion-extension, wrist abduction-adduction, and forearm pronation-supination [21]. Most of the aforementioned studies focused on proprioceptive training through specifically administering a set of tasks for improving sensorimotor function [11]. Moreover, protocols implemented for assessing and improving position sense in upper limbs considered two-dimensional space and administered isolated movements mostly in the transverse plane [10, 12–20] and sagittal plane [22–25]. As a large number of activities of daily living require moving the upper-limbs in three-dimensional (3D) trajectories [26], there is a need for rehabilitation protocols that require participants to practice movements in 3D space.

With the goal of improving limb position sense in 3D space, this study investigates the short-term effects of training reaching movements and arm position sense through a robotic training protocol that employed delayed visual and haptic feedback. Our hypothesis was that end-position error, as a measure of arm position sense, would be improved within the training session. The rehabilitation protocol included two targets placed at locations that approximated part of the movements required for eating, a basic activity of daily living [27]. To the best of the authors’ knowledge, this is the first study to consider such a protocol in 3D space. Preliminary results with fewer participants were presented in [28].

## Methods

### Participants

Twelve healthy adults (32 ±11 years old; 6 females and 6 males) participated in the study, which took place in a research laboratory. Participants were excluded if they had a neurological condition and/or upper-extremity musculoskeletal injury. The Research Ethics Board of Simon Fraser University approved the study, and all participants provided informed written consent.

### Experimental setup and task

**Fig. 1:**
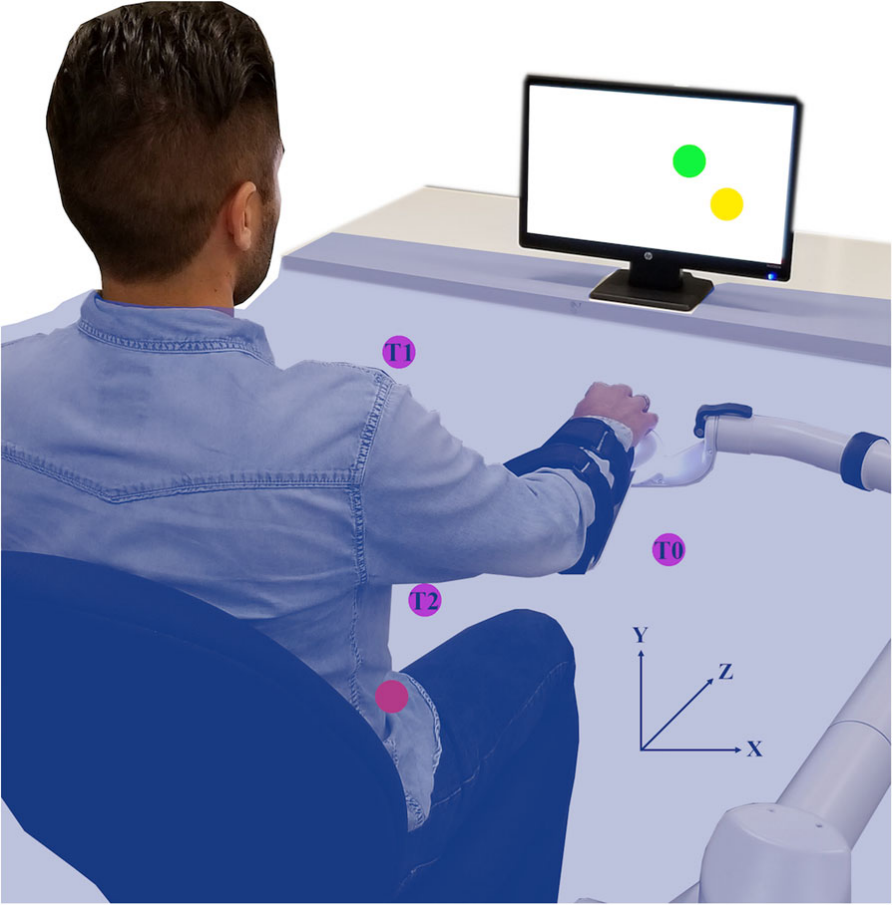
Experimental Setup. The system included an end-effector robotic arm and a computer screen. The participants’ hands and the robotic device were covered by a sheet of fabric (blue shadow). Participants began all reaching movements from their starting position (red sphere) and returned to this location after every reach. A target (yellow sphere) was presented on the computer screen and participants moved a cursor (green sphere) towards it. Three target locations (T0, T1, and T2) were employed (purple spheres)

The system (Fig. 1) was comprised of an end-effector robotic device for upper-limb rehabilitation (BURT, Barrett Technology, MA, USA) and a control computer running Ubuntu 16.04.3 (Canonical Group Limited, London, UK). The virtual task was developed in Unity 5.6.2 (Unity Technologies, CA, USA). During the task, participants were free to move the robot in any direction, as no forces were applied.

Participants were not able to see their arms nor the robot: they were asked to pass their head through the neck hole created in a sheet of fabric. Subsequently, the sheet was extended in every direction and leveled to the participants’ chin to cover their body below the neck as well as the robotic arm, while preventing participants’ arms or the robot from touching the sheet. The only manner in which participants received visual feedback about the task was via a computer screen placed 1.2 meters away from their seat. Participants’ knees were kept at 90 ^∘^ via a height-adjustable chair with no arm rests.

The task consisted of moving a cursor (Fig. 1, green sphere) towards a target (Fig. 1, yellow sphere) in three-dimensional space, as in our previous study [28]. The participants’ hand movements were recorded by the robot and mapped to cursor movements. There was no time limit for any of the reaching tasks, and participants were instructed to reach at their own preferred speed. In addition, participants were not constrained to reach the targets in a single movement.

At the beginning of the experimental session, the reach length of participants’ arms was recorded by the robot. This measurement, with assistance from the research team, was taken from the anatomical landmarks of participants’ ipsilateral hip to their knee. This length was employed to guarantee that all participants could reach to this distance, as in previous robotic rehabilitation studies [29, 30]. All targets locations were scaled to each participant’s reach length to ensure consistency between participants. The starting position before reaching to any target was always the same, close to each participant’s ipsilateral hip, as shown in Fig. 1.

**Fig. 2:**
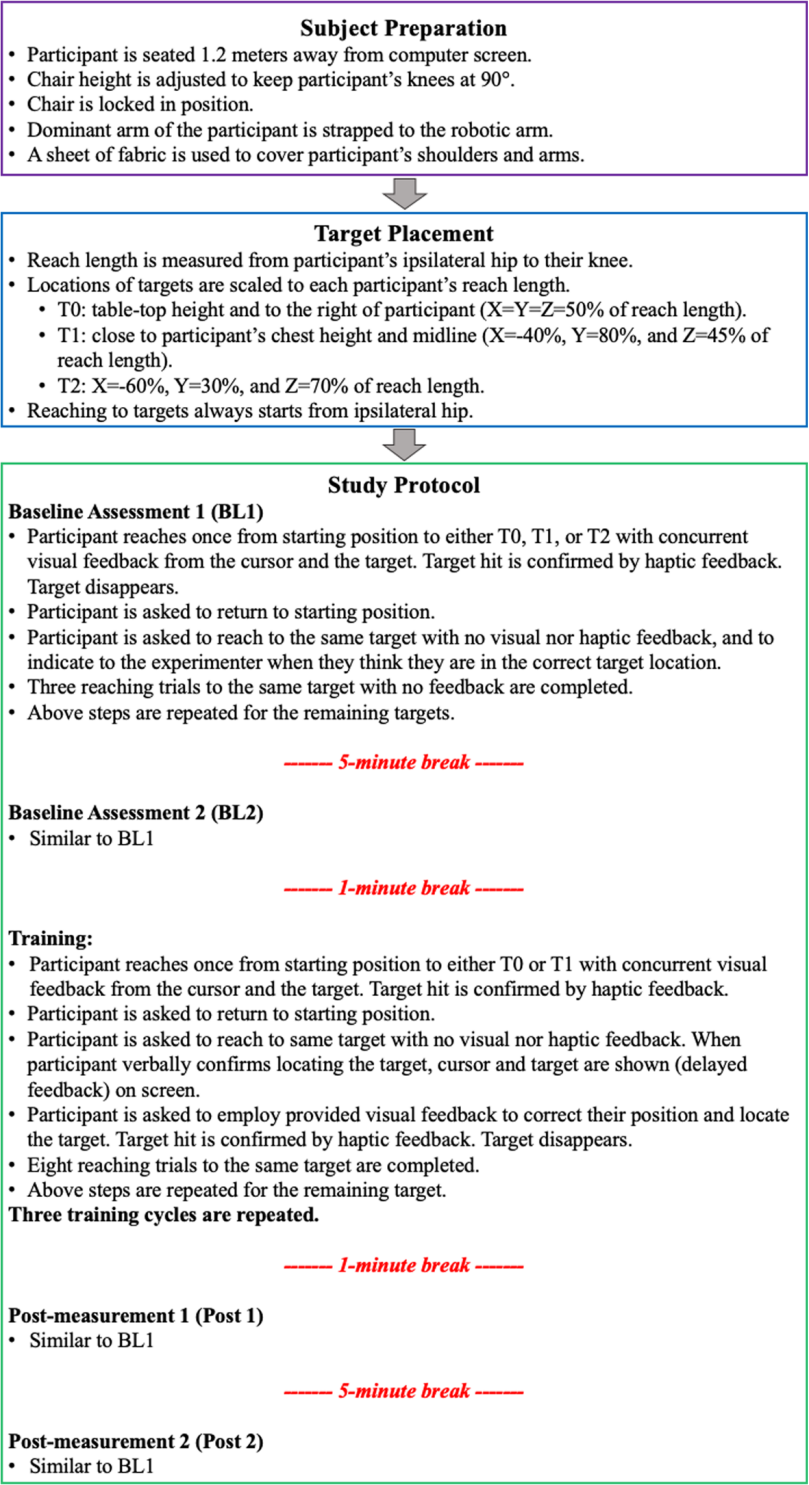
Experimental Protocol. The protocol was administered in each study session

In total, there were three target types (T0, T1, and T2). Two of the targets (T0 and T1) were placed at locations that approximated part of the movements required for eating, a basic activity of daily living [27]. T0 was placed at table-top height (X=Y=Z=50% of the reach length) and to the right of the participant, resembling the movement required for reaching for an eating utensil. T1 was placed close to participant’s chest height and midline (X=-40%, Y=80%, Z=45% of the reach length) to simulate part of the movement required to bring a utensil to the mouth. T2 was placed at a different consistent location (X=-60%, Y=30%, Z=70% of the reach length) for all participants to investigate if training to T0 and T1 could generalize to an untrained target. First and second blocks in Fig. 2 summarize the procedures followed to prepare participants and determine the location of targets.

The frame of reference was a left-handed coordinate system (Fig. 1). Medial-lateral hand movements were mapped to left-right cursor movements, superior-inferior hand movements were mapped to up-down cursor movements, and anterior-posterior hand movements were mapped to a change in cursor size, i.e., larger cursor meant closer to participant’s body while smaller cursor implied further away from body.

### Experimental session

The study protocol is summarized in the third block of Fig. 2. During the single experimental session, participants were asked to reach to targets, one at a time, with haptic feedback and concurrent or delayed visual feedback, or no visual/haptic feedback. Different types of feedback used in each step of the study are explained below:

- Haptic feedback was provided by the robotic devices via mechanical vibration (3 Nm, 1 s) at the robot’s end-effector when participants hit a target. The haptic feedback was used during the familiarization period and the training phase to indicate to participants that they were in the correct position and that the trial was over. It should be noted that during the familiarization period, this type of feedback was provided along with concurrent visual feedback. Participants received haptic and delayed visual feedback during the training phase.
- With *concurrent visual feedback*, the user-controlled cursor and the pre-calculated target were displayed on the computer screen for the entire duration of the reaching movement. This type of feedback was provided during the familiarization period as well as during the reference trial for the baseline, training, and post-measurements, as explained below.
- *Delayed visual feedback* was provided by displaying the cursor and the target only after the participants announced that they believed they were in the correct position. This type of feedback was provided during the training phase.
- In the *no visual/haptic feedback condition*, the computer screen did not display the target nor the cursor, and the robot end-effector did not provide haptic feedback. This condition was used during the baseline and post-measurement trials.

Participants were allowed to familiarize themselves with reaching with the robot and moving to the targets (T0, T1, and T2) at the beginning of the experimental session. During this familiarization episode, participants were able to move freely, and the cursor and all targets were displayed simultaneously on screen. In addition, participants were instructed to reach to all targets to become accustomed to the visuomotor transformation between their 3D arm movements and their visualization in 2D. Two baseline measurement blocks, separated by a 5-minute break, were employed to ensure that any observed improvement was in fact a result of the training and not because participants were exposed multiple times to the same visuomotor task. For the baseline blocks (Fig. 2), participants were asked, as the reference trial, to reach once to a randomly presented target (T0, T1, or T2) with concurrent visual feedback (cursor and target shown on screen). When participants hit the target, they received haptic confirmation (vibration) from the robot, and the target disappeared, indicating that the reaching trial was over. Subsequently, they were asked to reach to the same target without any visual or haptic feedback and to inform the experimenter when they thought they were in the correct target location. The experimented would then mark the trial as complete. Three reaching trials to the same target without visual or haptic feedback were completed, before the whole process was repeated with a new target. After every reaching movement, participants were asked to move back to their starting position. Two post-measurement blocks, separated by a 5-minute break, were employed to investigate how long the short-term effects of training would last. The post-measurement blocks followed the same structure and number of reaching movements as the baseline measurement blocks.

During the three blocks of training trials (Fig. 2), participants were asked to reach once to a randomly presented target (T0 or T1) with concurrent visual and haptic feedback, as the reference trial. Afterwards, they were asked to reach to this target without visual or haptic feedback, however, different to the baseline and post-measurement trials, when participants indicated that they were in the correct position, the visual feedback would be turned back on (delayed feedback), and participants would be given an opportunity to correct their position and hit the target, receiving haptic and visual confirmation. Participants performed eight training trials with delayed feedback to one of the targets before repeating the procedure with the other one. Three blocks of training trials were repeated with delayed visual feedback to each target,, for a total of 24 movements. This number was chosen based on previous literature [21] and a pilot study with one participant. The total number of reaching movements, including all blocks with concurrent, delayed and no feedback, performed in the single session was 102. On average, the experimental session lasted 36 (SD:6) minutes.

### Data analysis

The primary outcome measure was the three-dimensional error (Euclidean distance) between the cursor’s end-position and target. As exploratory measures, we investigated the maximum hand velocity, the participants’ reaching time, defined as the length of time during which the hand velocity was greater than 10% of the maximum velocity, and the end-position error in X, Y, and Z directions.

Factorial Repeated Measures ANOVAs were employed to test for differences in the primary outcome measure as well as the reaching time and maximum velocity. A within-subject factor of assessment type (BL1, BL2, Post1, and Post2), and a within-subject factor of target (T0, T1, and T2) were included in the analysis. The trials that were employed in the analysis were the ones in which the participant did not receive visual nor haptic feedback and returned to a remembered position. To explore the effects of reaching direction (X, Y, and Z) on end-position error, we performed an analysis with within-subject factors of direction, assessment and target. Departures from sphericity were adjusted using Greenhouse-Geisser correction, adjusted degrees of freedom are shown with decimals. For post-hoc tests, Bonferroni correction was employed, and corrected *p* values are presented. In the “Results” section, the standard error is shown after the ± symbol, unless otherwise indicated. All tests were conducted in SPSS Statistics v24.0 (IBM Corp., NY, USA).

## Results

**Fig. 3:**
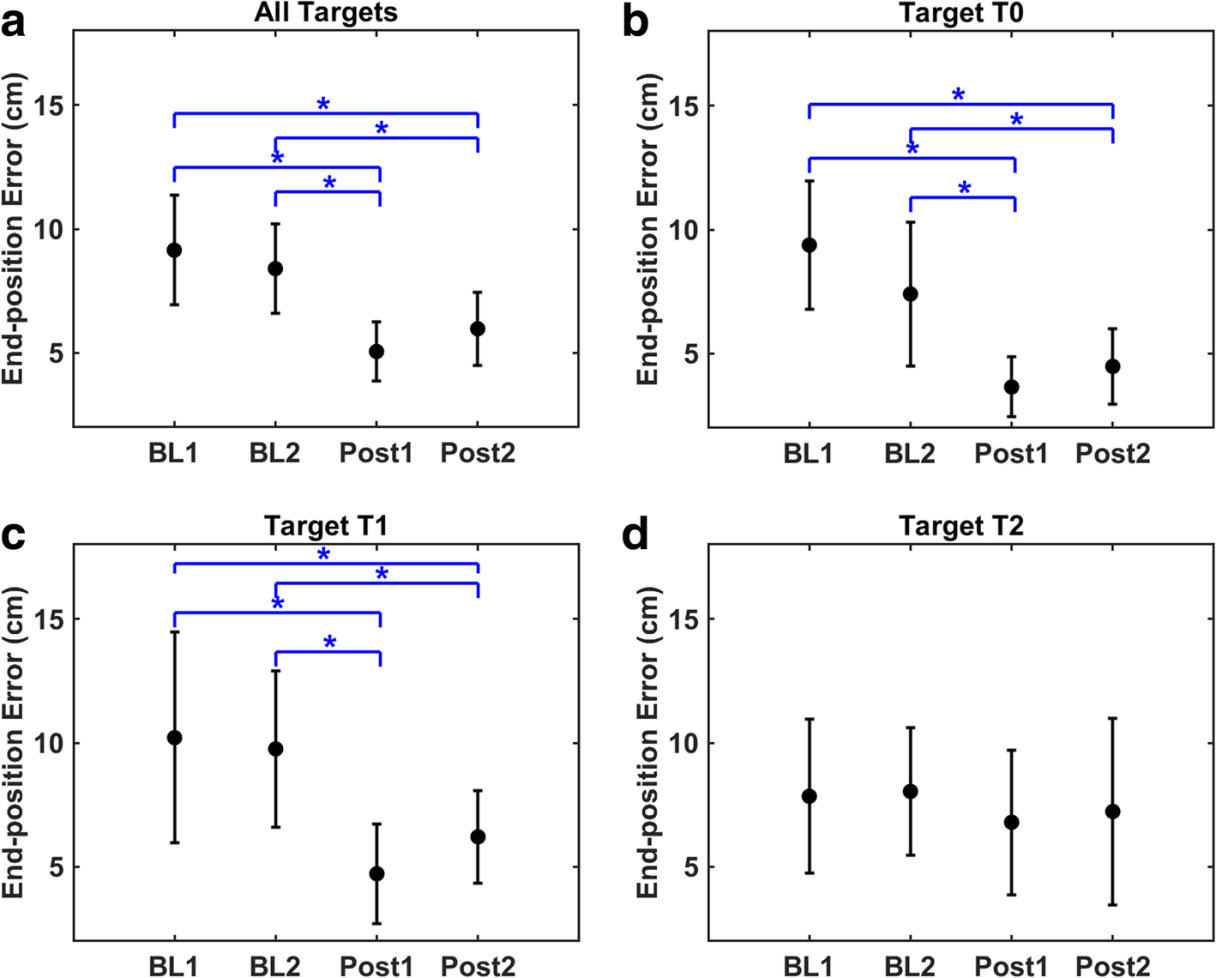
End-position error. Means and standard deviations displayed. The symbol * indicates statistical significance (*p <0.05). BL: baseline, Post: post-measurement

For the end-position error (Fig. 3a), the main effect of assessment type was statistically significant (F(1.9,20.9)=18.824, *p*<0.001). When all targets were combined, the average error was reduced by 44.71% (*p*=0.001) and 39.81% (*p*=0.001) when comparing Post1 to BL1 and BL2 trials, respectively; and 34.67% (*p*=0.017) and 28.88% (*p*=0.006) when comparing Post2 to BL1 and BL2 trials, respectively. Main results are summarized in Table 1. The main effect for target was not statistically significant (F(2,22)=1.752, *p*=0.197). The interaction effect for end-position error was statistically significant (F(6,66)=5.145, *p*<0.001).

**Table 1:** End-position error: change in the average value ±standard error is reported. A negative sign shows improvement in the measured value. The symbol * indicates statistical significance (*p <0.05). BL1 vs BL2 and Post1 vs Post2 were not significantly different in any of the shown cases. BL: baseline, Post: post-measurement

Measure	Target	BL1 vs Post1	BL1 vs Post2	BL2 vs Post1	BL2 vs Post2
End-position		-4.09 ±0.74	-3.17 ±0.83	-3.34 ±0.6	-2.43 ±0.55%
error (cm)	All	t(11)=5.545*	t(11)=3.83*	t(11)=5.568*	t(11)=4.437*
		-5.73 ±0.95	-4.9 ±0.71	-3.75 ±0.96	-2.92 ±0.69
	T0	t(11)=6.012*	t(11)=6.853*	t(11)=3.908*	t(11)=4.215*
		-5.49 ±1.1	-4.0 ±1.19	-5.03 ±0.93	-3.55 ±1.04
	T1	t(11)=5.006*	t(11)=3.345*	t(11)=5.382*	t(11)=3.394*
		-1.05 ±1.14	-0.61 ±1.23	-1.24 ±0.63	-0.81 ±0.79
	T2	t(11)=0.919	t(11)=0.5	t(11)=1.981	t(11)=1.018

For T0 (Fig. 3b), the average error was reduced by 61.07% (*p*=0.001) and 50.69% (*p*=0.015) when comparing Post1 to BL1 and BL2 trials, respectively; and 52.21% (*p*<0.001) and 39.46% (*p*=0.009) when comparing Post2 to BL1 and BL2 trials, respectively.

For T1 (Fig. 3c), the average error was reduced by 53.75% (*p*=0.002) and 51.6% (*p*=0.001) when comparing Post1 to BL1 and BL2 trials, respectively; and 39.17% (*p*=0.039) and 36.35% (*p*=0.036) when comparing Post2 to BL1 and BL2 trials, respectively.

For T2 (Fig. 3d), although not statistically significant, the average error was reduced by 13.37% (*p*=1.0) and 15.47% (*p*=0.439) when comparing Post1 to BL1 and BL2 trials, respectively; and 7.84% (*p*=1.0) and 10.07% (*p*=1.0) when comparing Post2 to BL1 and BL2 trials, respectively.

For the reaching time, the main effect of assessment type was not statistically significant (F(1.5,16.6)=3.438, *p*=0.067) nor the interaction between the factors of assessment and target (F(1.7,19.1)=2.067, *p*=0.158). The effect of target was significant (F(1.1,12.6)=7.012, *p*=0.018), with T0 having reduced times when compared to T1 (*Δ*=0.89 ±0.31s, t(11)=2.884, *p*=0.045) and T2 (*Δ*=0.41 ±0.11s, t(11)=3.78, *p*=0.009). For the maximum velocity, the main effect of assessment type was not statistically significant (F(1.5,16.6)=3.419, *p*=0.068) nor was the interaction between the factors of assessment and target (F(6,66)=1.193, *p*=0.321). The effect of target was significant (F(2,22)=32.946, *p*<0.001), with T0 having higher maximum velocities when compared to T1 (*Δ*=6.79 ±0.87cm/s, t(11)=7.812, *p*=0.045) and T2 (*Δ*=3.26 ±0.92cm/s, t(11)=3.545, *p*=0.014); and T2 having higher values than T1 (*Δ*=3.53 ±0.71cm/s, t(11)=4.998, *p*=0.001).

**Fig. 4:**
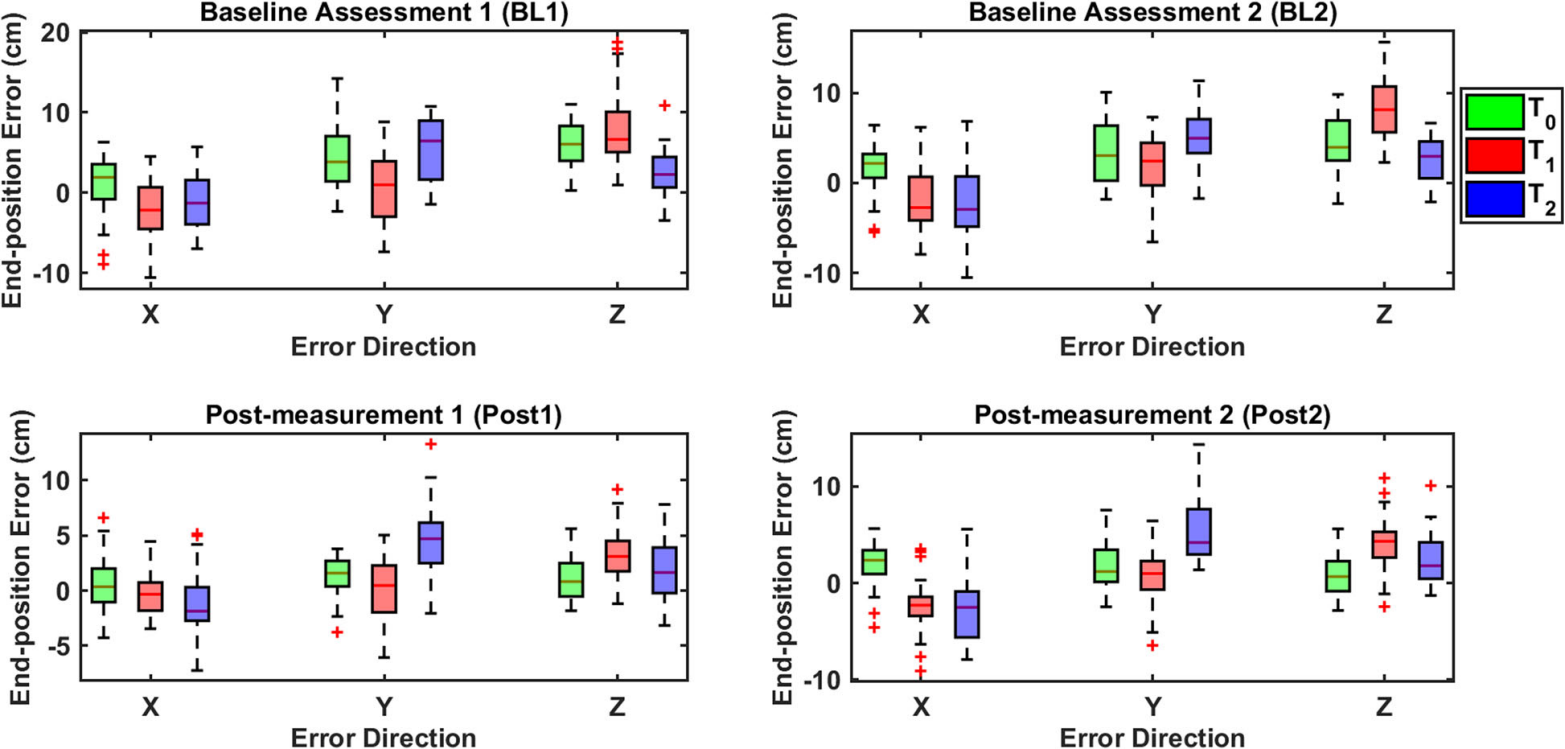
End-position error in each direction. The end-position error is shown for each direction and for each target across all reaches by all participants in baseline assessments and post-measurements. BL: baseline, Post: post-measurement

Figure 4 shows the end-position error in each direction and for each target across all reaches by all participants in baseline assessments and post-measurements. For the end-position direction analysis, the main effect of assessment type was statistically significant (F(2,22.1)=16.726, *p*<0.001), as well as the interaction between assessment and direction (F(6,66)=4.126, *p*=0.001), direction and target (F(4,44)=14.656, *p*<0.001), and assessment and target (F(6,66)=4.044, *p*=0.002). In the following, we describe the results of the post-hoc tests of the interaction terms. When all targets were combined (assessment-direction interaction), for the X direction, the error from BL2 to Post1 was reduced (*Δ*=1.08 ±0.26 cm, t(11)=4.086, *p*=0.011), and no consistent overshooting or undershooting was observed. In most cases for all targets, the hand position in the Z direction was underestimated so that the end-position was further from the body than desired. The training protocol significantly reduced the amount of overshooting in the Z direction, and the end-position error was reduced when comparing Post1 to BL1 (*Δ*=3.2 ±0.55 cm, t(11)=5.837, *p*=0.001) and BL2 trials (*Δ*=2.77 ±0.45 cm, t(11)=6.153, *p*<0.001); and when comparing Post2 to BL1 (*Δ*=2.83 ±0.67 cm, t(11)=4.222, *p*=0.009) and BL2 (*Δ*=2.39 ±0.47, t(11)=5.039, *p*=0.002) trials. When all assessments were combined (direction-target interaction), for T1 the Z direction had larger errors than the X (*Δ*=3.28 ±0.66 cm, t(11)=4.944, *p*=0.001) and Y (*Δ*=3.17 ±0.82 cm, t(11)=3.874, *p*=0.008) directions; for T2 the Y direction had larger errors than the Z direction (*Δ*=2.37 ±0.76 cm, t(11)=3.142, *p*=0.028). For the assessment-target interaction (all directions combined), for T0 the end-position error was reduced when comparing Post1 to BL1 (*Δ*=2.74 ±0.49 cm, t(11)=5.544, *p*=0.001) and BL2 (*Δ*=1.96 ±0.55 cm, t(11)=3.543, *p*=0.028); and when comparing Post2 to BL1 (*Δ*=2.35 ±0.32 cm, t(11)=7.305, *p*<0.001) and BL2 (*Δ*=1.57 ±0.41 cm, t(11)=3.869, *p*=0.016). For T1, the end-position error was reduced when comparing Post1 to BL1 (*Δ*=2.61 ±0.59 cm, t(11)=4.408, *p*=0.006) and BL2 (*Δ*=2.39 ±0.51 cm, t(11)=4.73, *p*=0.004); and when comparing Post2 to BL1 (*Δ*=1.88 ±0.58 cm, t(11)=3.265, *p*<0.045).

## Discussion

In this work, we proposed a robotic training paradigm to improve arm position sense in three-dimensional space, which is the first of its kind, as previous studies have focused on planar movements [20, 31]. The overall end-position error for the trained targets (T0 and T1) was reduced by 51-52% after the training and was maintained to a reduced level of 36-39%, after a 5-minute break. In addition, the maximum velocity and reaching time did not show changes after the second baseline, indicating that the effects of the arm position sense training protocol mostly affected 3D hand end position rather than dynamic movement performance. Our results suggest that a single session of arm position training in 3D space, using delayed visual and haptic feedback, improved the end-position accuracy of healthy participants to trained target locations. Our findings provide support for the potential of acute training protocols in 3D space to modulate proprioceptive sense of the upper-limb. However, the interpretation of the current findings must also take into account the contributions of delayed visual feedback and improved motor control of the upper-limb to the potential improvements in arm position sense.

For instance, the remembered visual target location could have influenced our results. Since the current training paradigm involved a reach to the visual target and with concurrent feedback of arm position before the movement back to the same target without either feedback type, this could invariably influence subsequent reaching performance to the trained target locations. Indeed, humans are quite capable of reaching to remembered visual target locations [32, 33], as well as reaching without cursor feedback [34–36] with a degree of accuracy. Since previous studies have shown that humans use a combination of remembered visual information and proprioceptive information about hand position to accurately reach to a remembered target location [32, 33], it is entirely possible that this was the strategy of the participants in the current study. Our current study design and available data do not allow us to distinguish the weighted contribution of each of these sources of sensory information (i.e., visual and proprioceptive). As this was the first study of its kind to investigate the effects of visuomotor training of the upper-limbs to potentially enhance 3D arm position sense, we elected to utilize a training paradigm that involved the integration of visual information as an initial step. Future research should further investigate the contributions of remembered visual target location and proprioceptive information by manipulating the amount and timing of each type of sensory feedback.

Further, it is possible that our findings may have resulted from a recalibration of the visual-motor relationship. Several studies have demonstrated that the relationship of visual input and motor output information can be recalibrated, shown by using visuomotor rotation tasks in 2D [34–36] and 3D coordinates [37]. Although, the current task did not utilize such a visuomotor adaptation paradigm, these studies demonstrate the possibility that the current training paradigm may have induced an alteration in the visual-motor relationship following the upper-limb training. Specifically, since reaching movements were made in 3D space, which was translated to the visual feedback on a 2D vertically oriented screen, an alteration of the visuomotor relationship likely adapted to this novel environment. Future research could control for the influence of a potentially adapted visuomotor relationship in relation to a proprioceptive training paradigm similar to the current study.

In a previous 3-day robotic proprioceptive training study investigating wrist movements of healthy adults, it was found that a combination of haptic and vibrotactile feedback lead to improvements in joint position sense [21]. In a clinical population, a previous two-dimensional non-immersive virtual reality intervention found that people with stroke were able to improve their end-position accuracy to trained targets after a week of arm position sense training [20]. The results from these previous studies and our work provide supporting evidence for the use of technology-aided repetitive training to improve arm or joint position sense.

Although the goal of the current task was to have participants rely on proprioceptive information to place their arm close to the remembered target location, we cannot discount the contributing factors of improved delayed visual feedback and motor execution to guide arm movements to the target location. First, a plethora of work demonstrates that healthy adults have the ability to accurately gaze towards [38] and reach and point to remembered target locations at relatively short visual delays [38–40]. How exactly this information is stored and potentially remapped in the brain is not entirely clear, however, a previously visually acquired target may remain relatively ‘constant’ in our visual store of space [40, 41], even with intervening eye movements [38] and with multiple remembered visual target locations [39]. This previous work interprets this to be a remapping of remembered visual target location based upon intervening eye and head movements. Therefore, it is possible that improved arm position sense in our 3D robotic training paradigm was influenced by visuomotor remapping of the remembered trained target locations. Secondarily, we cannot discount that our training protocol to improve arm position sense will by default include the visuomotor control system, given proprioception sense of arm position is inherently linked to motor execution [11, 21]. A previous study utilized non-visual feedback robotic training of wrist movements relying on proprioceptive sense and vibratory haptic feedback to guide movements to demonstrate improvements in upper-limb position sense [21]. In the current study, training of the arm position sense involved previous visual feedback of a remembered target position, followed by active movement towards the target. Although obtained results did not show significant change in the maximum hand velocity or reaching time, it should be noted that the current study was not designed to assess changes in kinematic measures of movement but rather overall arm position sense in 3D space. As these factors were outside of the scope of the current study, future work could investigate the contribution of such kinematic variables in robotic assisted training of arm position sense.

The focus of this intervention was on the improvement of arm position sense as a result of robotic training. However, end-effector robotic devices and exoskeletons, could also be employed for the characterisation of proprioceptive impairment of clinical populations. In support of this concept, previous research suggests that the use of quantitative parameters measured from upper-limb position and kinesthetic matching tasks, could be used to complement current ordinal scales employed in clinical practice [8, 42, 43]. As such, future studies could employ proprioceptive robotic measurements to evaluate the results of sensorimotor training, provided by therapists and/or robots, as part of physical rehabilitation programs of populations with upper-limb deficits.

In our study we include an extra target (T2) that was not trained during the session. This was done to test if the effects of the training paradigm could generalize to other areas of the reachable three-dimensional workspace. On average, the end-position error was improved by ∼15% after the training, and by ∼10% after a 5-minute break. However, these changes were not statistically significant and were only observed as a trend. It would appear that even though some improvement was obtained after this short-term training, it was not enough to generalize to this untrained target. In addition, in the aforementioned robotic wrist proprioceptive training study [21], it was found that training goal-directed reaching movements did not generalize to an untrained continuous tracking task. As such, even in that higher dose study (504 reaching trials over 3 days), the generalization of proprioception training was not observed. Future studies should investigate which factors (e.g., duration of trials, dosage, location of untrained targets, and/or the combination of visual and not visual trials) can lead to generalization after arm position training. Furthermore, characterizing the effects of singular or multi-modal types of feedback on promoting sense of position in upper-limbs and exploring the benefits of providing participants with game scores [30], if any, could be potential avenues for future robotic rehabilitation research.

In this study, we implemented the robotic training during a single session and with a short retention test (5 minutes). Future studies should investigate how learning and generalization is maintained in an extended intervention with longer retention tests, before such a protocol can be implemented in clinical practice. Another limitation of the study was that participants were only training with two targets that resembled a single activity of daily living (eating). However, other basic activities of daily living involve the use of the upper-limbs, such as the ones that are part of the personal care and dressing categories [44, 45]. Given that additional target positions and reachable workspaces could be implemented in similar end-effector robotic platforms, this could be an area of future research.

The current protocol was tested on young healthy adults, however, the next step could be to investigate if this robotic paradigm could be used in people with proprioceptive decline or impairment resulted from aging or neurological conditions, such as in older adults or people after stroke. In this next step, parsing out the visual and proprioceptive contributions to the observed improvements in end-position error would be paramount to design better training paradigms. In the case of people with stroke, as not only proprioceptive, but motor abilities can be affected [46], modifications to the current training paradigm might need to be implemented to allow people with motor disabilities to participate in similar interventions. For example, people after a stroke tend to compensate with their trunk when their reaching ability is impaired [47, 48], as such finding a robust manner to cover the arms and robot even when people move forward would be required. In addition, the visuomotor transformation between the participants’ 3D movements and the 2D displayed task could potentially make it more difficult for people with disabilities to complete our training paradigm. In this study, there was a consistent overshoot to targets in the Z direction, this underestimation might be partially due to the fact that the hand position in 3D was mapped to a 2D display: anterior-posterior hand movements were translated into changes in the size of the sphere-shaped cursor. Consequently, in the trials with concurrent visual feedback, in which the participant ‘learned’ the position of the target, adjusting the Z position of the hand could have been more challenging than locating the X and Y position of the target, for which the direction of cursor movements on screen matched that of the hand more closely. As such, a potential solution could be the use of immersive virtual reality goggles, as they would prevent participants from looking at their upper-body movements, while maintaining a 1:1 3D mapping between the robot movements and the displayed task. The immersive virtual reality environment might also help with reducing overshoots in the Z direction. In addition, people with upper-limb motor disabilities might require robotic assistance to perform arm movements to the displayed targets, which would call for the implementation of new control strategies for the robots.

## Conclusions and future work

This paper presented a three-dimensional robotic training protocol that aimed at improving upper-limb sense of position. Results suggest that young healthy adults benefited from improved position sense after a training session using delayed visual as well as haptic feedback to increase their awareness of the position of their arm in three-dimensional space. More specifically, it was observed that participants could reach to trained, but not untrained, targets with a significantly higher accuracy following the training phase. These results suggest that the current training protocol resulted in a trained-target specific improvement in arm position sense that is not generalizable to untrained arm positions. Altering the parameters of the training protocol, such as a longer training duration, multiple sessions, and a greater number of targets may aid in the improved generalization of our protocol.

## Data Availability

The datasets used and/or analyzed during the current study are available from the corresponding author on reasonable request.

## Abbreviations

BL1: Baseline Assessment 1
BL2: Baseline Assessment 2
Post1: Post-measurement 1
Post2: Post-measurement 2
T0: Target 0
T1: Target 1
T2: Target 2
2D: Two Dimensional
3D: Three Dimensional
